# Supplementary material for: Combination of Genomic Landsscape and 3D Culture Functional Assays Bridges Sarcoma Phenotype to Target and Immunotherapy
Source: Cells. 2023 Sep 4;12(17):2204. doi: 10.3390/cells12172204 (PMC10486752; doi:10.3390/cells12172204)
Supplement: Supplementary file 1 [file cells-12-02204-s001.zip › cells-2553708-supplementary.pdf]

cells

Review

Genomic Landscape and 3D Functional Assay Combination Bridges Sarcomas Phenotype to Target and Immunotherapy

Filomena de Nigris [1], Concetta Meo [1] and Wulf Palinski [2]

[1] Department of Precision Medicine, School of Medicine, University of Campania "Luigi Vanvitelli", 80138 Naples, Italy; concetta.meo@unicampania.it
[2] Department of Medicine, University of California San Diego, La Jolla, CA 92137, USA; w.palinski@ucsd.edu
* Correspondence: filomena.denigris@unicampania.it

Table S1. Morphologic Appearances, Immunohistochemical Markers and Molecular Alterations observed in selected sarcomas with simple karyotype

Sarcoma Type	Morphology and Immunohistochemical Markers	Chromosomal translocation	Molecular Fusion	Reference
Alveolar rhabdomyosarcoma	Round cell.	t(2;13)(q35;q14)	PAX3-FOXO1	[1]
	Positive for desmin, myogenin, MYOD1	t(1;13)(p36;q14)	PAX7-FOXO1	[1]
	(Myogenic markers)	t(2;2)(q35;p23)	PAX3-NCOA1	[2, 3]
		t(X;2)(q35;q13)	PAX3-AFX	[2]
Alveolar soft part sarcoma	Epithelioid/epithelial-like TFE3 positive	t(X;17)(p11.2;q25)	TFE3-ASPSCR1	[4, 5]
BCOR-rearranged sarcoma	Round cell	Inv(X)(p11p11)	BCOR-CCNB3	[6]
	Positive BCL6 corepressor	Inv(X)(p11p11)?	BCOR-MAML3	[7]
	CD99 variable	Inv(X)(p11p11)?	ZC3H7B-BCOR	[7, 8]
Sarcoma CIC-rearranged	Round cell	t(4;19)(q35;q13) or t(10;19)(q26;q13)	CIC-DUX4	[9]
	Positive ETV4, WT1 (CD99 partially in extent)	t(X;19)(q13;q13.3)	CIC-FOXO4	[10]
Clear cell sarcoma	n.d.	t(12;22)(q13;q12)	EWSR1-ATF1	[11]
	n.d.	t(2;22)(q32.3;q12)	CREB1-EWSR1	

Dermatofibrosarcoma protuberans	n.d.	t(17;22)(q22;q13)	COL1A1-PDGFB	[12]
Desmoplastic small round cell tumor	Round cell. Positive WT1 , CD99 partially	t(11;22)(p13;q12)	EWSR1-WT1	[12]
Endometrial stromal sarcoma, low grade	n.d.	t(7;17)(p15;q21)	JAZF1-SUZ12	
	n.d.	t(6;7)(p21;7p15)	PHF1-JAZF1	[13]
	n.d.	t(6;10)(p21;p11)	EPC1-PHF1	
	n.d.	t(1;6)(p34;p21)	MEAF6-PHF1	
	n.d	t(X;17)(p11;q21)	MBTD1-CXorf67	
Endometrial stromal sarcoma, high grade	n.d.	t(10;17)(q22;p13)	YWHAE-NUTM2	[14]
		t(X;22)(p11;q13)	ZC3H7B-BCOR	
Epithelioid hemangioendothelioma	Epithelioid/epithelial-like Positive ERG, CD31 (Vascular markers), CAMTA1 (90%) or TFE3 (5%)	t(1;3)(p36;q25)	WWTR1-CAMTA1	[15]
		t(X;11)(p11;q22)	YAP1-TFE3	[16]
Epithelioid sarcoma	Epithelioid/epithelial-like	Deletion 22q	SMARCB1	[17]
	Positive EMA/keratins and CD34 (50%)	t(8;22)(q22;q11)		
	Loss of SMARCB1 (INI1)	t(10;22)		
Ewing sarcoma	Round cell	t(11;22)(q24;q12)	EWSR1-FLI1	[18]
	Positive CD99 (membranous, diffuse) and NKX2-2	t(21;22)(q12;q12)	EWSR1-ERG	
		t(7;22)(p22;q12)	EWS-ETV1	
		t(17;22)(q12;q12)	EWS-ETV4	
		t(2;22)(q33;q12)	EWS-FEV	
Extraskeletal myxoid chondrosarcoma	Myxoid	t(9;22)(q22;q12)	EWSR1-NR4A3	[12]
	in 20% of cases: S100	t(9;17)(q22;q11)	TAF2N-NR4A3	
		t(9;15)(q22;q21)	TCF12-NR4A3	
		t(3;9)(q11;q22)	TFG-NR4A3	
		t(9;17)(q22;q11)	RBP56-NR4A3	
Inflammatory myofibroblastic tumor	Spindle cell	t(1;2)(q22;p23)	TPM3-ALK	[19-21]
	Positive ALK (50%), ROS1 (, 10%), smooth muscle markers	t(2;19)(p23;p13)	TPM4-ALK	
		t(2;17)(p23;q23)	CLTC-ALK	
		t(2;2)(p23;q13)	RANBP2-ALK	
	positive (subset)	t(2;2)(p23;q35)	ATIC-ALK	

		t(2;11)(p23;p15)	CARS-ALK	
		t(2;4)(p23;q21)	SEC31L1-ALK	
		t(2;12)(p23;p12)	PPFIBP1-ALK	
		?	RRBP1-ALK	
		?	TFG-ROS1	
		?	YWHAE-ROS1	
Low-grade fibromyxoid sarcoma	Myxoid	t(7;16)(q33;p11)	FUS-CREB3L2	[22,23]
	Positive MUC4	t(11;16)(p11;p11)	FUS-CREB3L1	
Mesenchymal chondrosarcoma		t(8;8)(q13;q21)	HEY1-NCOA2	[22]
Myxoid liposarcoma	Myxoid	t(12;16)(q13;p11)	FUS-DDIT3	[22]
		t(12;22)(q13;q12)	EWSR1-DDIT3	
Pseudomyogenic hemangioendothelioma	AP-1 transcription factor subunit (FOSB)	t(7;19)(q22;q13),	SERPINE1-FOSB	[24]
Sclerosing epithelioid fibrosarcoma	Epithelioid/epithelial-like	t(11;22)(p11;q12)	EWSR1-CREB3L1	[22,23]
		t(7;16)(q33;p11)	FUS-CREB3L2	
Solitary fibrous tumor	Spindle cell. Positive STAT6, CD34	Inv(12)(q13q13)	NAB2-STAT6	[25]
Synovial sarcoma	n.d.	t(X;22)(p11.23;q11)	SS18-SSX1	[26,27]
		t(X;18)(p11.21;q11)	SS18-SSX2	[26,27]
		t(X;18)(p11;q11)	SS18-SSX4	[28]

n.d. not determined

Table S2. Genetic alterations cause of complex karyotypes in sarcomas

Sarcoma Type	Genetic alterations	Genes affected	Mutation rate	Reference
Angiosarcoma	Mutations	*TP53, PTPRB*	66 %,26%	[29]
		PIK3K		[30]
	Overexpression	*VEGF*	80%	[29]
Chondrosarcoma	Mutations	*IDH*	50-80%	
		TP53	8%	[31]
Embryonal Rhabdomyosarcoma	Deletions	*CDKN2A/B*	23%	[32]
		RB	27%	[33]
	Activating Mutation	*FGR4*	20%	
		Ras family	42%	[32]
Fibrosarcoma	Amplifications	*MDM2*	Common	[34]
Leiomyosarcoma	Deletions	*PTEN*	57–69%	[35, 36]
		RB1	27–59%	[35, 36]
	Mutations	*TP53*	33–49%	[36-38]
		ATRX	17–26%	[36-38]
		MED12	21%	[38]
	Amplification	*MYOCD*	70%	[39]
Liposarcoma	Amplifications	*MDM2*	86-98%	[40,41],
		CDK4	58–88	[42]
		HMGA2	75–93%	[43]
		c-JUN	16-60%	[41]
MPNST	Mutations	*NF1*	87.5%	
		CDKN2A	75%	[42]
		TP53	40%	
		EED, SUZ12	Common	[43]
Osteosarcoma	Mutations	*TP53*	47-82%	[44, 45]
		RB1	29–47%	[44, 45]
		PIK3K		[46]
		DLG2	53%	[44]
		ATRX	29%	[44]

	Amplifications	*c-Myc*	39–42%	[47,48]
		CCNE1	33%	[48]
		RAD21	38%	[48]
		VEGFA	23%	[48]
		RUNX2	Common	[49,50]
Undifferentiated pleomorphic sarcoma	Deletions	*RB1*	30-35%	[51,52]

Reference Table S1

1. Sorensen, P. H.; Lynch, J. C.; Qualman, S. J.; Tirabosco, R.; Lim, J. F.; Maurer, H. M.; Bridge, J. A.; Crist, W. M.; Triche, T. J.; Barr, F. G. PAX3-FKHR and PAX7-FKHR gene fusions are prognostic indicators in alveolar rhabdomyosarcoma: a report from the children's oncology group. *J Clin Oncol* **2002**, *20* (11), 2672-2679. DOI: 10.1200/JCO.2002.03.137.
2. Sumegi, J.; Streblow, R.; Frayer, R. W.; Dal Cin, P.; Rosenberg, A.; Meloni-Ehrig, A.; Bridge, J. A. Recurrent t(2;2) and t(2;8) translocations in rhabdomyosarcoma without the canonical PAX-FOXO1 fuse PAX3 to members of the nuclear receptor transcriptional coactivator family. *Genes Chromosomes Cancer* **2010**, *49* (3), 224-236. DOI: 10.1002/gcc.20731.
3. Wachtel, M.; Dettling, M.; Koscielniak, E.; Stegmaier, S.; Treuner, J.; Simon-Klingenstein, K.; Buhlmann, P.; Niggli, F. K.; Schafer, B. W. Gene expression signatures identify rhabdomyosarcoma subtypes and detect a novel t(2;2)(q35;p23) translocation fusing PAX3 to NCOA1. *Cancer Res* **2004**, *64* (16), 5539-5545. DOI: 10.1158/0008-5472.CAN-04-0844.
4. Tsuji, K.; Ishikawa, Y.; Imamura, T. Technique for differentiating alveolar soft part sarcoma from other tumors in paraffin-embedded tissue: comparison of immunohistochemistry for TFE3 and CD147 and of reverse transcription polymerase chain reaction for ASPSCR1-TFE3 fusion transcript. *Hum Pathol* **2012**, *43* (3), 356-363. DOI: 10.1016/j.humpath.2011.05.004.
5. Aulmann, S.; Longerich, T.; Schirmacher, P.; Mechtersheimer, G.; Penzel, R. Detection of the ASPSCR1-TFE3 gene fusion in paraffin-embedded alveolar soft part sarcomas. *Histopathology* **2007**, *50* (7), 881-886. DOI: 10.1111/j.1365-2559.2007.02693.x.
6. Pierron, G.; Tirode, F.; Lucchesi, C.; Reynaud, S.; Ballet, S.; Cohen-Gogo, S.; Perrin, V.; Coindre, J. M.; Delattre, O. A new subtype of bone sarcoma defined by BCOR-CCNB3 gene fusion. *Nat Genet* **2012**, *44* (4), 461-466. DOI: 10.1038/ng.1107.
7. Specht, K.; Zhang, L.; Sung, Y. S.; Nucci, M.; Dry, S.; Vaiyapuri, S.; Richter, G. H.; Fletcher, C. D.; Antonescu, C. R. Novel BCOR-MAML3 and ZC3H7B-BCOR Gene Fusions in Undifferentiated Small Blue Round Cell Sarcomas. *Am J Surg Pathol* **2016**, *40* (4), 433-442. DOI: 10.1097/PAS.0000000000000591.
8. Kao, Y. C.; Sung, Y. S.; Zhang, L.; Jungbluth, A. A.; Huang, S. C.; Argani, P.; Agaram, N. P.; Zin, A.; Alaggio, R.; Antonescu, C. R. BCOR Overexpression Is a Highly Sensitive Marker in Round Cell Sarcomas With BCOR Genetic Abnormalities. *Am J Surg Pathol* **2016**, *40* (12), 1670-1678. DOI: 10.1097/PAS.0000000000000697.
9. Italiano, A.; Sung, Y. S.; Zhang, L.; Singer, S.; Maki, R. G.; Coindre, J. M.; Antonescu, C. R. High prevalence of CIC fusion with double-homeobox (DUX4) transcription factors in EWSR1-negative undifferentiated small blue round cell sarcomas. *Genes Chromosomes Cancer* **2012**, *51* (3), 207-218. DOI: 10.1002/gcc.20945.
10. Hung, Y. P.; Fletcher, C. D.; Hornick, J. L. Evaluation of ETV4 and WT1 expression in CIC-rearranged sarcomas and histologic mimics. *Mod Pathol* **2016**, *29* (11), 1324-1334. DOI: 10.1038/modpathol.2016.140.
11. Antonescu, C. R.; Tschernyavsky, S. J.; Woodruff, J. M.; Jungbluth, A. A.; Brennan, M. F.; Ladanyi, M. Molecular diagnosis of clear cell sarcoma: detection of EWS-ATF1 and MITF-M transcripts and histopathological and ultrastructural analysis of 12 cases. *J Mol Diagn* **2002**, *4* (1), 44-52. DOI: 10.1016/S1525-1578(10)60679-4.
12. Antonescu, C. R.; Dal Cin, P. Promiscuous genes involved in recurrent chromosomal translocations in soft tissue tumours. *Pathology* **2014**, *46* (2), 105-112. DOI: 10.1097/PAT.0000000000000049.
13. Antonescu, C. R.; Sung, Y. S.; Chen, C. L.; Zhang, L.; Chen, H. W.; Singer, S.; Agaram, N. P.; Sboner, A.; Fletcher, C. D. Novel ZC3H7B-BCOR, MEAF6-PHF1, and EPC1-PHF1 fusions in ossifying fibromyxoid tumors--molecular characterization shows genetic overlap with endometrial stromal sarcoma. *Genes Chromosomes Cancer* **2014**, *53* (2), 183-193. DOI: 10.1002/gcc.22132.
14. Hoang, L. N.; Aneja, A.; Conlon, N.; Delair, D. F.; Middha, S.; Benayed, R.; Hensley, M. L.; Park, K. J.; Hollmann, T. J.; Hameed, M. R.; et al. Novel High-grade Endometrial Stromal Sarcoma: A Morphologic Mimicker of Myxoid Leiomyosarcoma. *Am J Surg Pathol* **2017**, *41* (1), 12-24. DOI: 10.1097/PAS.0000000000000721.
15. Errani, C.; Zhang, L.; Sung, Y. S.; Hajdu, M.; Singer, S.; Maki, R. G.; Healey, J. H.; Antonescu, C. R. A novel WWTR1-CAMTA1 gene fusion is a consistent abnormality in epithelioid hemangioendothelioma of different anatomic sites. *Genes Chromosomes Cancer* **2011**, *50* (8), 644-653. DOI: 10.1002/gcc.20886.
16. Tanas, M. R.; Sboner, A.; Oliveira, A. M.; Erickson-Johnson, M. R.; Hespelt, J.; Hanwright, P. J.; Flanagan, J.; Luo, Y.; Fenwick, K.; Natrajan, R.; et al. Identification of a disease-defining gene fusion in epithelioid hemangioendothelioma. *Sci Transl Med* **2011**, *3* (98), 98ra82. DOI: 10.1126/scitranslmed.3002409.
17. Hornick, J. L.; Dal Cin, P.; Fletcher, C. D. Loss of INI1 expression is characteristic of both conventional and proximal-type epithelioid sarcoma. *Am J Surg Pathol* **2009**, *33* (4), 542-550. DOI: 10.1097/PAS.0b013e3181882c54.

18. Gamberi, G.; Cocchi, S.; Benini, S.; Magagnoli, G.; Morandi, L.; Kreshak, J.; Gambarotti, M.; Picci, P.; Zanella, L.; Alberghini, M. Molecular diagnosis in Ewing family tumors: the Rizzoli experience--222 consecutive cases in four years. *J Mol Diagn* **2011**, *13* (3), 313-324. DOI: 10.1016/j.jmoldx.2011.01.004.
19. Sholl, L. M.; Weremowicz, S.; Gray, S. W.; Wong, K. K.; Chirieac, L. R.; Lindeman, N. I.; Hornick, J. L. Combined use of ALK immunohistochemistry and FISH for optimal detection of ALK-rearranged lung adenocarcinomas. *J Thorac Oncol* **2013**, *8* (3), 322-328. DOI: 10.1097/JTO.0b013e31827db604.
20. Antonescu, C. R.; Suurmeijer, A. J.; Zhang, L.; Sung, Y. S.; Jungbluth, A. A.; Travis, W. D.; Al-Ahmadie, H.; Fletcher, C. D.; Alaggio, R. Molecular characterization of inflammatory myofibroblastic tumors with frequent ALK and ROS1 gene fusions and rare novel RET rearrangement. *Am J Surg Pathol* **2015**, *39* (7), 957-967. DOI: 10.1097/PAS.0000000000000404.
21. Hornick, J. L.; Sholl, L. M.; Dal Cin, P.; Childress, M. A.; Lovly, C. M. Expression of ROS1 predicts ROS1 gene rearrangement in inflammatory myofibroblastic tumors. *Mod Pathol* **2015**, *28* (5), 732-739. DOI: 10.1038/modpathol.2014.165.
22. Doyle, L. A.; Moller, E.; Dal Cin, P.; Fletcher, C. D.; Mertens, F.; Hornick, J. L. MUC4 is a highly sensitive and specific marker for low-grade fibromyxoid sarcoma. *Am J Surg Pathol* **2011**, *35* (5), 733-741. DOI: 10.1097/PAS.0b013e318210c268.
23. Doyle, L. A.; Wang, W. L.; Dal Cin, P.; Lopez-Terrada, D.; Mertens, F.; Lazar, A. J.; Fletcher, C. D.; Hornick, J. L. MUC4 is a sensitive and extremely useful marker for sclerosing epithelioid fibrosarcoma: association with FUS gene rearrangement. *Am J Surg Pathol* **2012**, *36* (10), 1444-1451. DOI: 10.1097/PAS.0b013e3182562bf8.
24. Walther, C.; Tayebwa, J.; Lilljebjorn, H.; Magnusson, L.; Nilsson, J.; von Steyern, F. V.; Ora, I.; Domanski, H. A.; Fioretos, T.; Nord, K. H.; et al. A novel SERPINE1-FOSB fusion gene results in transcriptional up-regulation of FOSB in pseudomyogenic haemangioendothelioma. *J Pathol* **2014**, *232* (5), 534-540. DOI: 10.1002/path.4322.
25. Doyle, L. A.; Vivero, M.; Fletcher, C. D.; Mertens, F.; Hornick, J. L. Nuclear expression of STAT6 distinguishes solitary fibrous tumor from histologic mimics. *Mod Pathol* **2014**, *27* (3), 390-395. DOI: 10.1038/modpathol.2013.164.
26. Kawai, A.; Woodruff, J.; Healey, J. H.; Brennan, M. F.; Antonescu, C. R.; Ladanyi, M. SYT-SSX gene fusion as a determinant of morphology and prognosis in synovial sarcoma. *N Engl J Med* **1998**, *338* (3), 153-160. DOI: 10.1056/NEJM199801153380303.
27. Ladanyi, M.; Antonescu, C. R.; Leung, D. H.; Woodruff, J. M.; Kawai, A.; Healey, J. H.; Brennan, M. F.; Bridge, J. A.; Neff, J. R.; Barr, F. G.; et al. Impact of SYT-SSX fusion type on the clinical behavior of synovial sarcoma: a multi-institutional retrospective study of 243 patients. *Cancer Res* **2002**, *62* (1), 135-140.
28. Skytting, B.; Nilsson, G.; Brodin, B.; Xie, Y.; Lundeberg, J.; Uhlen, M.; Larsson, O. A novel fusion gene, SYT-SSX4, in synovial sarcoma. *J Natl Cancer Inst* **1999**, *91* (11), 974-975. DOI: 10.1093/jnci/91.11.974.
29. Zietz, C.; Rossle, M.; Haas, C.; Sendelhofert, A.; Hirschmann, A.; Sturzl, M.; Lohrs, U. MDM-2 oncoprotein overexpression, p53 gene mutation, and VEGF up-regulation in angiosarcomas. *Am J Pathol* **1998**, *153* (5), 1425-1433. DOI: 10.1016/S0002-9440(10)65729-X.
30. Hollander, M. C.; Blumenthal, G. M.; Dennis, P. A. PTEN loss in the continuum of common cancers, rare syndromes and mouse models. *Nat Rev Cancer* **2011**, *11* (4), 289-301. DOI: 10.1038/nrc3037.
31. Amary, M. F.; Bacsi, K.; Maggiani, F.; Damato, S.; Halai, D.; Berisha, F.; Pollock, R.; O'Donnell, P.; Grigoriadis, A.; Diss, T.; et al. IDH1 and IDH2 mutations are frequent events in central chondrosarcoma and central and periosteal chondromas but not in other mesenchymal tumours. *J Pathol* **2011**, *224* (3), 334-343. DOI: 10.1002/path.2913.
32. Paulson, V.; Chandler, G.; Rakheja, D.; Galindo, R. L.; Wilson, K.; Amatruda, J. F.; Cameron, S. High-resolution array CGH identifies common mechanisms that drive embryonal rhabdomyosarcoma pathogenesis. *Genes Chromosomes Cancer* **2011**, *50* (6), 397-408. DOI: 10.1002/gcc.20864.
33. Dyson, N. J. RB1: a prototype tumor suppressor and an enigma. *Genes Dev* **2016**, *30* (13), 1492-1502. DOI: 10.1101/gad.282145.116.
34. Schmidt, H.; Taubert, H.; Wurl, P.; Kappler, M.; Lange, H.; Bartel, F.; Bache, M.; Holzhausen, H. J.; Hinze, R. Gains of 12q are the most frequent genomic imbalances in adult fibrosarcoma and are correlated with a poor outcome. *Genes Chromosomes Cancer* **2002**, *34* (1), 69-77. DOI: 10.1002/gcc.10036.
35. El-Rifai, W.; Sarlomo-Rikala, M.; Knuutila, S.; Miettinen, M. DNA copy number changes in development and progression in leiomyosarcomas of soft tissues. *Am J Pathol* **1998**, *153* (3), 985-990. DOI: 10.1016/S0002-9440(10)65640-4.
36. Chudasama, P.; Mughal, S. S.; Sanders, M. A.; Hubschmann, D.; Chung, I.; Deeg, K. I.; Wong, S. H.; Rabe, S.; Hlevnjak, M.; Zapatka, M.; et al. Integrative genomic and transcriptomic analysis of leiomyosarcoma. *Nat Commun* **2018**, *9* (1), 144. DOI: 10.1038/s41467-017-02602-0.
37. Yang, C. Y.; Liau, J. Y.; Huang, W. J.; Chang, Y. T.; Chang, M. C.; Lee, J. C.; Tsai, J. H.; Su, Y. N.; Hung, C. C.; Jeng, Y. M. Targeted next-generation sequencing of cancer genes identified frequent TP53 and ATRX mutations in leiomyosarcoma. *Am J Transl Res* **2015**, *7* (10), 2072-2081.
38. Makinen, N.; Aavikko, M.; Heikkinen, T.; Taipale, M.; Taipale, J.; Koivisto-Korander, R.; Butzow, R.; Vahteristo, P. Exome Sequencing of Uterine Leiomyosarcomas Identifies Frequent Mutations in TP53, ATRX, and MED12. *PLoS Genet* **2016**, *12* (2), e1005850. DOI: 10.1371/journal.pgen.1005850.
39. Perot, G.; Derre, J.; Coindre, J. M.; Tirode, F.; Lucchesi, C.; Mariani, O.; Gibault, L.; Guillou, L.; Terrier, P.; Aurias, A. Strong smooth muscle differentiation is dependent on myocardin gene amplification in most human retroperitoneal leiomyosarcomas. *Cancer Res* **2009**, *69* (6), 2269-2278. DOI: 10.1158/0008-5472.CAN-08-1443.
40. Hostein, I.; Pelmus, M.; Aurias, A.; Pedeutour, F.; Mathoulin-Pelissier, S.; Coindre, J. M. Evaluation of MDM2 and CDK4 amplification by real-time PCR on paraffin wax-embedded material: a potential tool for the diagnosis of atypical lipomatous tumours/well-differentiated liposarcomas. *J Pathol* **2004**, *202* (1), 95-102. DOI: 10.1002/path.1495.

41. Saada-Bouzid, E.; Burel-Vandenbos, F.; Ranchere-Vince, D.; Birtwisle-Peyrottes, I.; Chetaille, B.; Bouvier, C.; Chateau, M. C.; Peoc'h, M.; Battistella, M.; Bazin, A.; et al. Prognostic value of HMGA2, CDK4, and JUN amplification in well-differentiated and dedifferentiated liposarcomas. *Mod Pathol* **2015**, *28* (11), 1404-1414. DOI: 10.1038/modpathol.2015.96.
42. Brohl, A. S.; Kahen, E.; Yoder, S. J.; Teer, J. K.; Reed, D. R. The genomic landscape of malignant peripheral nerve sheath tumors: diverse drivers of Ras pathway activation. *Sci Rep* **2017**, *7* (1), 14992. DOI: 10.1038/s41598-017-15183-1.
43. Lee, W.; Teckie, S.; Wiesner, T.; Ran, L.; Prieto Granada, C. N.; Lin, M.; Zhu, S.; Cao, Z.; Liang, Y.; Sboner, A.; et al. PRC2 is recurrently inactivated through EED or SUZ12 loss in malignant peripheral nerve sheath tumors. *Nat Genet* **2014**, *46* (11), 1227-1232. DOI: 10.1038/ng.3095.
44. Chen, X.; Bahrami, A.; Pappo, A.; Easton, J.; Dalton, J.; Hedlund, E.; Ellison, D.; Shurtleff, S.; Wu, G.; Wei, L.; et al. Recurrent somatic structural variations contribute to tumorigenesis in pediatric osteosarcoma. *Cell Rep* **2014**, *7* (1), 104-112. DOI: 10.1016/j.celrep.2014.03.003.
45. Kovac, M.; Blattmann, C.; Ribi, S.; Smida, J.; Mueller, N. S.; Engert, F.; Castro-Giner, F.; Weischenfeldt, J.; Kovacova, M.; Krieg, A.; et al. Exome sequencing of osteosarcoma reveals mutation signatures reminiscent of BRCA deficiency. *Nat Commun* **2015**, *6*, 8940. DOI: 10.1038/ncomms9940.
46. Albers, J.; Danzer, C.; Rechsteiner, M.; Lehmann, H.; Brandt, L. P.; Hejhal, T.; Catalano, A.; Busenhart, P.; Goncalves, A. F.; Brandt, S.; et al. A versatile modular vector system for rapid combinatorial mammalian genetics. *J Clin Invest* **2015**, *125* (4), 1603-1619. DOI: 10.1172/JCI79743.
47. Gamberi, G.; Benassi, M. S.; Bohling, T.; Ragazzini, P.; Molendini, L.; Sollazzo, M. R.; Pompetti, F.; Merli, M.; Magagnoli, G.; Balladelli, A.; et al. C-myc and c-fos in human osteosarcoma: prognostic value of mRNA and protein expression. *Oncology* **1998**, *55* (6), 556-563. DOI: 10.1159/000011912.
48. Sayles, L. C.; Breese, M. R.; Koehne, A. L.; Leung, S. G.; Lee, A. G.; Liu, H. Y.; Spillinger, A.; Shah, A. T.; Tanasa, B.; Straessler, K.; et al. Genome-Informed Targeted Therapy for Osteosarcoma. *Cancer Discov* **2019**, *9* (1), 46-63. DOI: 10.1158/2159-8290.CD-17-1152.
49. Won, K. Y.; Park, H. R.; Park, Y. K. Prognostic implication of immunohistochemical Runx2 expression in osteosarcoma. *Tumori* **2009**, *95* (3), 311-316. DOI: 10.1177/030089160909500307.
50. Gupta, S.; Ito, T.; Alex, D.; Vanderbilt, C. M.; Chang, J. C.; Islamdoust, N.; Zhang, Y.; Nafa, K.; Healey, J.; Ladanyi, M.; et al. RUNX2 (6p21.1) amplification in osteosarcoma. *Hum Pathol* **2019**, *94*, 23-28. DOI: 10.1016/j.humpath.2019.09.010.
51. Chibon, F.; Mairal, A.; Freneaux, P.; Terrier, P.; Coindre, J. M.; Sastre, X.; Aurias, A. The RB1 gene is the target of chromosome 13 deletions in malignant fibrous histiocytoma. *Cancer Res* **2000**, *60* (22), 6339-6345.
52. Mertens, F.; Fletcher, C. D.; Dal Cin, P.; De Wever, I.; Mandahl, N.; Mitelman, F.; Rosai, J.; Rydholm, A.; Sciot, R.; Tallini, G.; et al. Cytogenetic analysis of 46 pleomorphic soft tissue sarcomas and correlation with morphologic and clinical features: a report of the CHAMP Study Group. Chromosomes and MorPhology. *Genes Chromosomes Cancer* **1998**, *22* (1), 16-25. DOI: 10.1002/(sici)1098-2264(199805)22:1<16::aid-gcc3>3.0.co;2-a.

Table S3. Clinical trials with target therapy in sarcoma (source: www.clinicaltrials.gov, accessed on July, 2023).

Clinical trials with CDKi therapy in sarcoma					
**NCT Number**	**Study Title**	**Sarcoma Type**	**Target**	**Drug Name**	**Study Status/ Phase**
NCT05905341	Study of PF-07224826, as a Single Agent or in Combination With Endocrine Therapy in Participants With Breast Cancer and Other Advanced Solid Tumors.	Liposarcoma	CDK2/4/6	PF-07224826	Not yet Recruiting/ Phase 1
NCT05655598	TAS-116 Plus Palbociclib in Breast and Rb-null Cancer	Soft Tissue Sarcoma	CDK4/6	TAS-116 Plus Palbociclib	Not yet Recruiting/ Phase 1
NCT05496569	TQB3616 Capsules in the Treatment of Dedifferentiated Liposarcoma	Dedifferentiated Liposarcoma	CDK4/6	TQB3616	Not yet Recruiting/ Phase 2

NCT05159518	A Study of PRT2527 in Participants With Advanced Solid Tumors	Sarcoma	CDK9	PRT2527	Recruiting/ Phase 1
NCT03242382	Trial of Palbociclib in Second Line of Advanced Sarcomas with CDK4 Overexpression	Advanced Sarcomas with CDK4 Overexpression	CDK4/6	Palbociclib	Recruiting/ Phase 2
NCT04040205	Abemaciclib for Bone and Soft Tissue Sarcoma with Cyclin-Dependent Kinase (CDK) Pathway Alteration	Advanced sarcoma (CS, OS, soft tissue sarcoma except LPS) with CDK pathway alteration	CDK4/6	Abemaciclib	Recruiting/ Phase 2
NCT03604783	Phase 1, First-in-human Study of Oral TP-1287 in Patients with Advanced Solid Tumors	Sarcoma	CDK9	TP-1287	Recruiting/ Phase 1
NCT04941274	Abemaciclib in Patients With HIV-associated and HIV-negative Kaposi Sarcoma	Kaposi Sarcoma	CDK4/6	Abemaciclib	Recruiting/ Phase 1-2
NCT02644460	Abemaciclib in Children with DIPG or Recurrent/Refractory Solid Tumors	Ewing Sarcoma, Rhabdomyosarcoma, Osteosarcoma Recurrent/Refractory,	CDK4/6	Abemaciclib	Recruiting/ Phase 1
NCT04557449	Study to Test the Safety and Tolerability of PF-07220060 in Participants with Advance Solid Tumors	Liposarcoma	CDK4/6	PF-07220060 administered as a single agent and then in combination with endocrine therapy	Recruiting/ Phase 1-2
NCT04438824	Palbociclib and INCMGA00012 in People with Advanced Liposarcoma	Well-differentiated/ Dedifferentiated Liposarcoma	CDK4/6	Palbociclib and INCMGA00012	Recruiting/ Phase 2
NCT03784014	Molecular Profiling of Advanced Soft-tissue Sarcomas	Advanced Soft-tissue Sarcomas	CDK	Nilotinib, Ceritinib, Capmatinib, Lapatinib, Trametinib, Trametinib and Dabrafenib, Olaparib and Durvalumab,	Recruiting/ Phase 3

				Palbociclib, Glasdegib, TAS-120	
NCT05252416	(VELA) Study of BLU-222 in Advanced Solid Tumors	Carcinosarcoma	CDK 2	BLU-222, Carboplatin, Ribociclib, Fulvestrant	Recruiting/ Phase 1-2
NCT01209598	PD0332991 (Palbociclib) in Patients With Advanced or Metastatic Liposarcoma	Advanced/Metastatic Liposarcoma	CDK4/6	Palbociclib	Completed/ Phase 2
NCT04129151	Palbociclib + Ganitumab In Ewing Sarcoma	Ewing Sarcoma	CDK4/6 + IGF1R	Palbociclib + Ganitumab	Completed/ Phase 2
NCT03709680	Study Of Palbociclib Combined with Chemotherapy In Pediatric Patients With Recurrent/Refractory Solid Tumors	Recurrent/Refractory Ewing Sarcoma, Rhabdomyosarcoma in children	CDK4/6 + chemotherapy	Palbociclib + Temozolomide + Irinotecan	Recruiting/ Phase 2
NCT02897375	Palbociclib With Cisplatin or Carboplatin in Advanced Solid Tumors	Sarcoma	CDK4/6 + chemotherapy	Palbociclib + Cisplatin or Carboplatin	Active, not recruiting
NCT02846987	Study of Abemaciclib in Dedifferentiated Liposarcoma	Sarcoma/ Dedifferentiated Liposarcoma	CDK4/6	Abemaciclib	Active, not recruiting
NCT02784795	A Study of LY3039478 in Participants With Advanced or Metastatic Solid Tumors	Soft Tissue Sarcoma	Notch + CDK4/6	LY3039478 + Abemaciclib	Completed/ Phase 1
NCT03009201	Ribociclib and Doxorubicin in Treating Patients With Metastatic or Advanced Soft Tissue Sarcomas That Cannot Be Removed by Surgery	Metastatic or Advanced, Unresectable Soft Tissue Sarcomas	CDK4/6	Ribociclib + Doxorubicin	Active, not recruiting
NCT03114527	Phase II Trial of Ribociclib and Everolimus in Advanced Dedifferentiated Liposarcoma (DDL) and Leiomyosarcoma (LMS)	Soft Tissue Sarcoma	CDK4/6 + mTOR	Ribociclib + Everolimus	Active, not recruiting
NCT02343172	Study of Safety and Efficacy of HDM201 in Combination With	Liposarcoma	HDM2 + CDK4/6	HDM201 + Ribociclib	Completed/ Phase 1

	LEE011 in Patients With Liposarcoma				
NCT03310879	Study of the CDK4/6 Inhibitor Abemaciclib in Solid Tumors Harboring Genetic Alterations in Genes Encoding D-type Cyclins or Amplification of CDK4 or CDK6	Cancer with Genetic Alterations in D-type Cyclins or CDK4/6 Amplification	CDK4/6	Abemaciclib	Recruiting/ Phase 2
NCT01907607	Efficacy and Safety of PD-0332991 in Advanced Gastrointestinal Stromal Tumors Refractory to Imatinib and Sunitinib	Advanced Gastrointestinal Stromal Tumors	CDK4/6	Palbociclib	Completed/ Phase 2
NCT02571829	A Phase II Study Assessing Efficacy & Safety of Ribociclib in Patients With Advanced Well/Dedifferentiated Liposarcoma	Liposarcoma/ Soft Tissue Sarcoma	CDK4/6	Ribociclib	Phase 2
**Clinical trials with target P53 and MDM2/X interactions in sarcoma**					
**NCT Number**	**Study Title**	**Sarcoma Type**	**Target**	**Drug Name**	**Study Status/ Phase**
NCT02432963	Vaccine Therapy and Pembrolizumab in Treating Patients With Solid Tumors That Have Failed Prior Therapy	Solid Tumors/ Soft Tissue Sarcoma	P53	Modified Vaccinia Virus Ankara Vaccine Expressing p53 and Pembrolizumab	Active, not recruiting/ Phase 1
NCT01386502	CT-011 and p53 Genetic Vaccine for Advanced Solid Tumors	Sarcoma	P53	p53: 264-272 peptide and CT-011	Withdrawn
NCT03727789	CBL0137 in Treating Patients With Advanced Extremity Melanoma or Sarcoma	Advanced Sarcoma of the Extremity/ Recurrent Sarcoma of the Extremity	Active p53, inhibits the histone chaperone and inhibits NF-κB	Complex-targeting Curaxin CBL0137	Recruiting/ Phase 1
NCT00676910	A Research Study of JNJ-26854165 to Determine the Safety and Dose in Patients With Advanced Stage or Refractory Solid Tumors	Neoplasm/Sarcoma	P53 Activator	JNJ-26854165	Completed/ Phase 1
NCT03217266	Navtemadlin and Radiation Therapy in Treating Patients With Soft Tissue Sarcoma	Resectable Soft Tissue Sarcoma	MDM2	Navtemadlin and Radiation Therapy	Active, not recruiting/ Phase 1

NCT02343172	Study of Safety and Efficacy of HDM201 in Combination With LEE011 in Patients With Liposarcoma	Liposarcoma	Selective inhibitor of the p53-Mdm2 interaction	HDM201 and LEE011	Completed/ Phase 1
NCT05180695	HDM201 and Pazopanib in Patients With P53 Wild-type Advanced/Metastatic Soft Tissue Sarcomas	Advanced Soft-tissue Sarcoma|Metastatic Soft-tissue Sarcoma	Selective inhibitor of the p53-Mdm2 interaction	HDM201 and Pazopanib	Recruiting/ Phase 1-2
NCT03611868	A Study of APG-115 in Combination With Pembrolizumab in Patients With Metastatic Melanomas or Advanced Solid Tumors	Unresectable or Metastatic Melanoma or Advanced Solid Tumors|P53 Mutation|MDM2 Gene Mutation	MDM2/p53 inhibitor	APG-115+ Pembrolizumab	Recruiting/ Phase 1-2
NCT01143740	A Study of RO5045337 in Patients With Liposarcomas Prior to Debulking Surgery	Sarcoma	Selective inhibitor of the p53-Mdm2 interaction	RO5045337	Completed/ Phase 1
NCT00559533	A Study of RO5045337 [RG7112] in Patients With Advanced Solid Tumors	Neoplasm/Sarcoma	Selective inhibitor of the p53-Mdm2 interaction	RO5045337	Completed/ Phase 1
NCT00223184	Genetic Characterisation of High-grade Paediatric Osteosarcomas	Paediatric Osteosarcomas			
NCT00898755	Collecting and Storing Tissue From Young Patients With Cancer	Rhabdomyosarcoma|Soft Tissue Sarcoma			
**Clinical trials with target PI3K or PI3K/Akt/mTOR pathway in sarcoma**					
**NCT Number**	**Study Title**	**Sarcoma Type**	**Target**	**Drug Name**	**Study Status/ Phase**
NCT01833169	BKM120 for Patients With PI3K-activated Tumors	PI3K Pathway Activated Tumors	PI3K Pathway	BKM120	Completed/ Phase 1
NCT01357330	Oral SAR245408 (XL147) and Oral MSC1936369B in Patients With Locally Advanced or Metastatic Solid Tumors	Solid Tumors/ Sarcoma	PI3K Pathway	SAR245408 (XL147) and MSC1936369Bi	Completed/ Phase 2

NCT00907205	A Dose Escalation Study of SF1126, a PI3 Kinase (PI3K) Inhibitor, Given By Intravenous (IV) Infusion in Patients With Solid Tumors	Solid Tumors/ Sarcoma	PI3K Pathway	SF1126	Completed/ Phase 1
NCT00996892	Safety, Tolerability and Pharmacokinetics of Cobimetinib in Combination With Pictilisib in Patients With Locally Advanced or Metastatic Solid Tumors	Solid Tumors/ Sarcoma	PI3K Pathway	Cobimetinib + Pictilisib	Terminated/ Phase 1
NCT01343498	Study of PI3 Kinase/mTOR Inhibitor BEZ235 Twice Daily for Advanced Solid Tumors	Advance Solid Tumors/ Sarcoma	PI3K/mTOR	BEZ235	Completed/ Phase 1
NCT00600275	A Phase I/II Study of BGT226 in Adult Patients With Advanced Solid Malignancies Including Patients With Advanced Breast Cancer	Solid Tumors/ Sarcoma	PI3K/mTOR	BGT226	Completed/ Phase 1-2
NCT00485719	Study of the Safety and Pharmacokinetics of XL765 (SAR245409) in Adults With Solid Tumors	Solid Tumors/ Sarcoma	PI3K/mTOR	XL765 (SAR245409)	Completed/ Phase 1
NCT03213678	Samotolisib in Treating Patients With Relapsed or Refractory Advanced Solid Tumors, Non-Hodgkin Lymphoma, or Histiocytic Disorders With TSC or PI3K/MTOR Mutations	Soft-tissue Sarcoma/Advance d Solid Tumors	PI3K/mTOR	Samotolisib	Active, not recruiting/ Phase 2
NCT02987959	Study of TAK-228 (MLN0128) in Soft Tissue Sarcomas	Soft-tissue Sarcoma	mTOR	TAK-228	Terminated/ Phase 2
NCT03660930	Nab-Sirolimus and Pazopanib Hydrochloride in Treating Patients With Advanced Nonadipocytic Soft Tissue Sarcomas	Soft-tissue Sarcoma	mTOR	Nab-Sirolimus and Pazopanib	Active, not recruiting/ Phase 2
NCT03190174	Nivolumab (Opdivo®) Plus ABI-009 (Nab-rapamycin) for Advanced Sarcoma and Certain Cancers	Advanced Sarcoma	mTOR	Nab-rapamycin + Nivolumab	Completed/ Phase 1-2
NCT00093080	Study of AP23573/MK-8669 (Ridaforolimus), A Mammalian Target of Rapamycin (mTOR)	Advanced Sarcoma	mTOR	Ridaforolimus	Completed/ Phase 2

	Inhibitor, in Participants With Advanced Sarcoma (MK-8669-018 AM1)(COMPLETED)				
NCT01684449	Gemcitabine Plus Rapamycin Versus Gemcitabine to Treat Advanced Soft Tissue Sarcoma	Advanced Soft Tissue Sarcoma	mTOR	Gemcitabine + Rapamycin	Completed/ Phase 1-2
NCT00949325	Safety and Efficacy Study of Torisel and Liposomal Doxorubicin for Patients With Recurrent Sarcoma	Sarcoma	mTOR	Temsirolimus plus liposomal Doxorubicin	Completed/ Phase 1-2
NCT01614795	Cixutumumab and Temsirolimus in Treating Younger Patients With Recurrent or Refractory Sarcoma	Recurrent or Refractory Sarcoma	mTOR	Cixutumumab and Temsirolimus	Completed/ Phase 2
NCT00996346	Phase I/II Study of Irinotecan and Temsirolimus in Patients With Refractory Sarcomas	Sarcoma	mTOR	Irinotecan and Temsirolimus	Terminated/ Phase 1
NCT03114527	Phase II Trial of Ribociclib and Everolimus in Advanced Dedifferentiated Liposarcoma (DDL) and Leiomyosarcoma (LMS)	Soft-tissue Sarcoma	mTOR	Ribociclib and Everolimus	Active, not recruiting/ Phase 2
NCT02584647	PLX3397 Plus Sirolimus in Unresectable Sarcoma and Malignant Peripheral Nerve Sheath Tumors	Sarcoma	mTOR	PLX3397 + Sirolimus	Recruiting/ Phase 1-2
NCT02821507	Sirolimus and Cyclophosphamide in Metastatic or Unresectable Myxoid Liposarcoma and Chondrosarcoma	Liposarcoma and Chondrosarcoma	mTOR	Sirolimus and Cyclophospham ide	Completed/ Phase 2
